# Barriers and Facilitators Affecting Patient Portal Implementation from an Organizational Perspective: Qualitative Study.

**DOI:** 10.2196/jmir.8989

**Published:** 2018-05-11

**Authors:** Laura Kooij, Wim G Groen, Wim H van Harten

**Affiliations:** ^1^ Division of Psychosocial Research and Epidemiology Netherlands Cancer Institute Amsterdam Netherlands; ^2^ Department of Health Technology and Services Research University of Twente Enschede Netherlands; ^3^ Rijnstate Hospital Arnhem Netherlands

**Keywords:** patient portals, health information technology, attitude of health personnel

## Abstract

**Background:**

The number of patient portals is rising, and although portals can have positive effects, their implementation has major impacts on the providing health care institutions. However, little is known about the organizational factors affecting successful implementation. Knowledge of the specific barriers to and facilitators of various stakeholders is likely to be useful for future implementations.

**Objective:**

The objective of this study was to identify the barriers to and facilitators of patient portal implementation facing various stakeholders within hospital organizations in the Netherlands.

**Methods:**

Purposive sampling was used to select hospitals of various types. A total of 2 university medical centers, 3 teaching hospitals, and 2 general hospitals were included. For each, 3 stakeholders were interviewed: (1) medical professionals, (2) managers, and (3) information technology employees. In total, 21 semistructured interviews were conducted using the Grol and Wensing model, which describes barriers to and facilitators of change in health care practice at 6 levels: (1) innovation; (2) individual professional; (3) patient; (4) social context; (5) organizational context; and (6) economic and political context. Two researchers independently selected and coded quotes by applying this model using a (deductive) directed content approach. Additional factors related to technical and portal characteristics were added using the model of McGinn et al, developed for implementation of electronic health records.

**Results:**

In total, we identified 376 quotes, 26 barriers, and 28 facilitators. Thirteen barriers and 12 facilitators were common for all stakeholder groups. The facilitators’ *perceived usefulness* (especially less paperwork) was mentioned by all the stakeholders, followed by subjects’ *positive attitude*. The main barriers were *lack of resources* (namely, lack of staff and materials), *financial difficulties* (especially complying with high costs, lack of reimbursements), and *guaranteeing privacy and security* (eg, strict regulations). Both similarities and differences were found between stakeholder groups and hospital types. For example, managers and information technology employees mainly considered *guaranteeing privacy and security* as a predominant barrier. *Financial difficulties* were particularly mentioned by medical professionals and managers.

**Conclusions:**

Patient portal implementation is a complex process and is not only a technical process but also affects the organization and its staff. Barriers and facilitators occurred at various levels and differed among hospital types (eg, *lack of accessibility*) and stakeholder groups (eg, *sufficient resources*) in terms of several factors. Our findings underscore the importance of involving multiple stakeholders in portal implementations. We identified a set of barriers and facilitators that are likely to be useful in making strategic and efficient implementation plans.

## Introduction

Patient-centeredness is an important element of high-quality care: effective communication between patients and their health care professionals, and information access can both contribute considerably to this [1]. According to the Institute of Medicine, “patients should have unfettered access to their own medical information” [2] to support them in taking control of their health (eg, using medical information to make informed health-related decisions) [2]. Information technology (IT) can play an important role in improving access to this information [3], and it also improves the participation of patients in their own care [4]. In health care, an increasingly popular way to facilitate this is by using patient portals [5]. Patient portals can be defined as “applications which are designed to give the patient secure access to health information and allow secure methods for communication and information sharing” [6], as well as for administrative purposes [7], and are mostly provided by a single health care institution [6,8]. These portals are often connected to the electronic health record (EHR) of an institution—defined as tethered patient portals [9]—to provide access to patients’ medical information [3,10-12]. Some institutions allow patient portals to facilitate communication between patients and health care professionals [3,6,12], view their appointments and provide patient education [11,13], share information [12], request for repeat medication prescriptions [3], and provide tailored feedback [11,13]. Patient portals may have a range of functionalities that enable information exchange (such as having access to the EHR), which in turn may facilitate and improve the communication between the patient and the health care professional [11,14]. Previous research showed that patients are especially satisfied with access to information from the EHR and the list of their appointments [11]. Portal use can also have a positive effect on self-management of conditions [15-18], communication between patients and providers, quality of care [16,17] and participation in treatment [17]. Patient empowerment can also be improved; the accessibility of information can especially contribute to “patients’ knowledge” and their “perception of autonomy and being respected” [19]. On the other hand, effects on health outcomes are reported to be mixed [6]. In summary, patient portals can be important as they provide patients with access to their own medical information, enable interaction with their health care professionals [8], and aim to involve patients in their own care processes [1].

Although patient portals can have positive effects and may develop into a standard element of care [20], their implementation has major impacts on health care institutions as it often involves a complex change in an organization [1]. This can be affected by multiple factors at the micro (eg, “individuals”), meso (eg, “resources”), and macro (eg, “sociopolitical context”) levels [21]. Several implementation models are available, such as “The Consolidated Framework for Implementation Research (CFIR),” which is used in many studies as a guiding framework [22-24]. CFIR consists of 5 levels at which barriers and facilitators can occur during implementation: (1) technology-related factors (eg, “adaptability,” “complexity,” and “cost”); (2) outer setting (eg, “policy and incentives”); (3) inner setting (eg, “resources”); (4) process (eg, “engagement of stakeholders”); and (5) individual health professionals (eg, “individual’s knowledge”). In this model, patients are part of the “outer setting,” suggesting that the CFIR framework is aimed primarily at institutions [24]. Another example is the “Fit between Individuals, Tasks, and Technology” (FITT) framework, which is aimed at the adoption of IT [25]. The comprehensive model of Grol and Wensing [26] summarizes the barriers to and facilitators of change in health care practice at 6 levels: (1) innovation; (2) individual professional; (3) patient; (4) social context; (5) organizational context; and (6) economic and political context. McGinn et al [21] argue that the consideration of various stakeholder opinions can contribute to successful implementations. However, previous research mainly focused on perceptions of single stakeholder groups regarding patient portal implementation, such as physicians [27] or nurses [28]. This highlights the importance of identifying the opinions of many stakeholders during patient portal implementation. Furthermore, it remains unclear which factors are important in accomplishing change in the various groups [26].

Previous research focused on patient involvement in developing patient portals [5,14], but little is yet known about organizational factors that facilitate or hinder patient portal implementation [6]. Such knowledge is essential because the number of portals is rising. In the Netherlands, in 2017, more than 25% of hospitals provided patients with access to a patient portal, whereas this was under 10% in 2015 [29]. Comprehensive information can provide a framework for upcoming patient portal implementations, or other eHealth applications, in hospitals. The objective of this study was, therefore, to identify the barriers and facilitators among the various stakeholders within hospital organizations in the Netherlands regarding the implementation of tethered patient portals.

## Methods

### Sampling Procedure

Purposive sampling was used to select hospitals of the 3 different types existing in the Netherlands. In total, 2 university medical centers (UMCs), 3 teaching and 2 general hospitals (including one collaborative oncology hospital comprising 3 general hospitals) were included. Hospitals were selected by means of convenience sampling using the authors’ network or by Web searching, and hospitals in various phases of implementation (contemplation, preparation, or implementation) were included. Contact persons in the hospitals were approached by phone or email. Snowball sampling was used for the selection of respondents, meaning that we informed the contact persons about the objective of the study and also asked them for contact information for 3 stakeholders, including (1) medical professionals (doctor or nurse practitioners [Advanced Practice Registered Nurses]) [30], (2) managers, and (3) IT employees.

**Table 1:** Barriers and facilitators at various levels of Grol and Wensing.

Levels of Grol and Wensing [26]	Examples of barriers and facilitators
Innovation: patient portal	Accessibility, attractiveness, and credibility
Individual professional	Knowledge, attitude, and motivation to change
Patient	Knowledge, skills, and attitude
Social context	Opinions of colleagues, culture of the networks, and collaboration
Organizational context	Organization of care processes, staff, and resources
Economic and political context	Financial arrangements, regulations, and policies

If the contact person belonged to one of these groups, they were also asked to participate. Once the stakeholders had agreed to participate, an interview was scheduled with each person individually. In total, 8 hospitals were approached, of which 7 agreed to participate, and 21 subjects participated in the study. No ethical review is needed for this type of study. All participants were informed about the purpose of the study, and participation was voluntary. Verbal consent for audio recording the interviews was obtained for every participant. All data were analyzed and presented anonymously.

### Data Collection Procedure

The interviews were conducted by the first author (LK). A few days before the interview, each participant received a confirmation email suggesting a scheduled date and time. A document was attached describing the objectives of the study and a topic list for the interview. We also added our own definition of a typical patient portal: “a personal digital environment, facilitated by a health care institution, for example a hospital. Patients need to login to the portal to get access to, for example, their medical file (with results), patient information and appointments. Patients can also fill in questionnaires and receive personalized advice regarding, for example, quality of life and physical activity.” We used a semistructured interview that was structured by applying the comprehensive model of Grol and Wensing [26] that summarizes the barriers to and facilitators of change in health care practice. This model describes 6 levels at which barriers and facilitators can occur: (1) innovation: patient portal; (2) individual professional; (3) patient; (4) social context; (5) organizational context; and (6) economic and political context. All these barriers and facilitators are described in Table 1.

All interviews were performed by telephone and lasted for, on average, 20 min. Participants were first asked for their consent to make audio recordings of the interviews. Then, the purpose of the interview was introduced, and subjects were asked if they received the introductory email. This email was then briefly discussed such that the subjects were aware of the topics to be discussed. After that, questions were asked about participants’ characteristics, such as their age and work experience. To make sure an unambiguous definition of a patient portal was used, participants were asked what their definition of a patient portal was, and if necessary, it was complemented with our definition. Then, we asked them about their perceived barriers to and facilitators of patient portal implementation at all 6 levels [26]. If necessary, for example, if the question was unclear, the interviewer provided examples (and these were also sent per email). At the end of the interview, the participants were asked to suggest additional topics or issues, if any, that had not yet been covered. The interviews were in Dutch, and the questions in Multimedia Appendix 1 are translations.

### Data Analysis

The first author transcribed all interviews verbatim. Two researchers (LK and WG) independently selected text fragments that reflected a barrier to or facilitator of portal implementation and coded the transcripts in Excel according to the model of Grol and Wensing [26]. A directed content approach was used, which is mainly a deductive approach as a pre-existing model is used for coding [31]. If quotes did not fit into the Grol and Wensing model [26], we looked for categories from the McGinn model [21], which was developed for implementation of EHRs. These models have considerable overlap, but the Grol and Wensing model [26] mainly covers socio-dynamic factors, whereas the McGinn model [21] also covers technical and portal characteristics. For the remaining quotes we created new categories, which is an inductive approach. To enhance clarity and unambiguity of the categories, we renamed them to better reflect the nature of being a barrier or a facilitator. A complete overview of the categories is presented in Multimedia Appendix 2. Coding was discussed between LK and WG until consensus was reached. Saturation of the data was checked by the first author by assessing (post hoc) the percentage of new categories appearing with the analysis of every subsequent hospital.

## Results

### Characteristics of the Subjects

In total, we interviewed 21 stakeholders from 7 hospitals. We included 3 from each hospital including medical professionals (n=7), managers (n=7), and IT employees (n=7). The stakeholder group labeled medical professionals consisted of medical specialists (n=4) and nurse practitioners (n=3). The group of managers included a medical director (n=1), hospital division or department managers (n=5), and a project manager (n=1). IT employees were application specialists or managers (n=3), an IT manager (n=1), an IT architect and information manager (n=1), and a patient portal project manager (n=2). Mean age was 44.8 years (SD 6.7; range 25-61) and 57% (12/21) were female. We included 6 respondents (6/21, 29%) from UMCs, 9 respondents (9/21, 43%) from teaching hospitals, and 6 (6/21, 29%) from general hospitals. Participants’ work experience varied from 6 years or less (10/21, 48%) to more than 21 years (3/21, 14%). An overview of participants’ characteristics is listed in Table 2.

### Barriers to and Facilitators of Patient Portal Implementation

In total, we selected 376 quotes and identified 26 barriers and 28 facilitators. The results are presented according to the 6 levels of the Grol and Wensing model [26]. The full list of all barriers and facilitators—including the number of subjects for each stakeholder group—is presented in Multimedia Appendix 3. After the inclusion of 7 hospitals (using purposive sampling), we analyzed the data saturation. The data were found to be saturated, meaning that after analyzing the first 6 hospitals, no new categories emerged from the transcripts of the final hospital. We therefore did not include further hospitals.

Due to the high number of identified barriers and facilitators, only those common to all stakeholder groups (medical professionals, managers, and IT employees) are presented here. To demonstrate the similarities and differences between stakeholder groups and between hospitals types, their most mentioned barriers and facilitators are presented as well.

### Barriers and Facilitators Common to All Stakeholder Groups

In total, 13 barriers and 12 facilitators (Table 3) were identified that were common to all stakeholder groups. The most relevant barriers and facilitators for each level are presented based on the number of subjects (and percentage of the total subjects) and are highlighted in *italics.* Quotes are used to illustrate the barriers and facilitators for each level that were mentioned by the majority of the subjects.

**Table 2:** Participants’ characteristics (N=21).

Characteristics		n (%)
**Gender**		
	Female	12 (57)
	Male	9 (43)
**Age (years)**		
	20-29	3 (14)
	30-39	3 (14)
	40-49	7 (33)
	50-59	6 (29)
	>60	2 (10)
**Hospital**		
	University medical centers	6 (29)
	Teaching hospital	9 (42)
	General hospital	6 (29)
**Work experience in current position in organization (years)**		
	≤5	10 (48)
	6-10	3 (14)
	11-15	1 (5)
	16-20	4 (19)
	≥21	3 (14)

**Table 3:** Barriers to and facilitators of patient portal implementation mentioned by all stakeholder groups and ranked by number of subjects.

Barriers and facilitators			Stakeholders, n (%)			
			Medical professionals (n=7)	Managers (n=7)	IT employees (n=7)	Total (n=21)
**Innovation: patient portal**							
	**Barriers**					
		Guaranteeing privacy and security	1 (14)	5 (71)	5 (71)	11 (52)
		Lack of accessibility	2 (29)	4 (57)	3 (43)	9 (43)
		Lack of perceived usefulness	4 (57)	1 (14)	2 (29)	7 (33)
	**Facilitators**					
		Perceived usefulness	7 (100)	7 (100)	7 (100)	21 (100)
		Perceived ease of use	2 (29)	2 (29)	1 (14)	5 (24)
		Attractiveness	1 (14)	1 (14)	2 (29)	4 (19)
		Participation of end users during implementation	1 (14)	1 (14)	1 (14)	3 (14)
**Individual professional**						
	**Facilitators**					
		Positive attitude	3 (43)	7 (100)	3 (43)	13 (62)
		Motivation to change	4 (57)	2 (29)	2 (29)	8 (38)
		Having knowledge	1 (14)	2 (29)	2 (29)	5 (24)
**Patient**						
	**Barriers**					
		Lack of sufficient eHealth literacy	4 (57)	5 (71)	4 (57)	13 (62)
**Social context**						
	**Barriers**					
		Negative attitude or opinion of medical professionals	4 (57)	3 (43)	1 (14)	8 (38)
	**Facilitators**					
		Positive attitude or opinion of medical professionals	1 (14)	2 (29)	2 (29)	5 (24)
**Organizational context**						
	**Barriers**					
		Lack of resources	4 (57)	5 (71)	6 (86)	15 (71)
		Lack of time and increased workload	4 (57)	3 (43)	1 (14)	8 (38)
		Innovation-averse culture	1 (14)	4 (57)	1 (14)	6 (29)
		Lack of suitable specialist staff	1 (14)	2 (29)	3 (43)	6 (29)
		Adjusting organization of care processes is difficult	2 (29)	1 (14)	2 (29)	5 (24)
		Structure of the organization	2 (29)	1 (14)	2 (29)	5 (24)
		Change in task and new responsibilities	1 (14)	1 (14)	2 (29)	4 (19)
	**Facilitator**					
		Management support	2 (29)	3 (43)	3 (43)	8 (38)
		Communication to promote the portal	1 (14)	4 (57)	1 (14)	6 (29)
		Innovation-oriented culture	2 (29)	2 (29)	1 (14)	5 (24)
**Economic and political context**						
	**Barrier**					
		Financial difficulties	5 (71)	6 (86)	3 (43)	14 (67)
	**Facilitator**					
		Facilitating laws and regulations	1 (14)	2 (29)	1 (14)	4 (19)

#### Innovation: Patient Portal

##### Barriers

*Lack of perceived usefulness*, *lack of accessibility*, and *guaranteeing privacy and security* were identified as barriers for portal implementation. Important reasons related to the privacy and security were the regulations, the availability of privacy-sensitive information on the portal, and the requirements for a safe login. The login or authorization method used in the Netherlands—the so-called digital identity DigiD with additional text messaging verification—was mentioned very frequently and can therefore be considered a major barrier. This DigiD login consists of a username and password of the user’s own choice and provides citizens with access to hundreds of government websites in the Netherlands [32]:

The security is a barrier for both the organization, and the implementation of the portal, as well for patients. The moment we secure the data according to the law and regulations, we notice that the use is not what it could be.

Manager, university medical center

Due to the privacy and security aspects, accessibility of the portal is increasingly becoming a limitation, and this was mainly because of the requirement for a DigiD login. Subjects mentioned lack of perceived usefulness because the portal implementation can lead to discord and practical difficulties. In addition, the portal only provides information for one health care institution, so patients do not have a complete overview of their health information.

##### Facilitators

*Perceived usefulness, attractiveness, perceived ease of use*, and *participation of end users* during implementation were seen as facilitators for implementation. All subjects (n=21) see *perceived usefulness* as a facilitator because the implementation of a patient portal could result in fewer consults, less paperwork, higher quality of care, and financial savings. Also for patients, multiple benefits were listed, including more involvement in their treatment, more transparency, and better accessibility of information:

It saves a lot of paperwork and hassles. It sounds ideal to me. Currently patients receive so many paper documents that they don’t have an overview anymore. If we centralize this on a portal it will be more clear for them.

Medical professional, general hospital

A good project team and the *participation of the end users during implementation* —both patients and hospital staff—can be beneficial because their input can be used to make adjustments during the development phase. *Perceived ease of use* and specifically the design of the portal can facilitate portal use, and the *attractiveness* was widely considered to be a requirement.

#### Individual Professional

##### Facilitators

No barriers were common for all the stakeholder groups. However, all groups see *motivation to change*, *knowledge*, and their own *positive attitude* as a facilitator:

I am very happy that we are starting with this development and that we, I think, are taking positive steps for the healthcare in the Netherlands.

Manager, teaching hospital

#### Patient

##### Barriers

Only barriers were anticipated for patients (common to all stakeholder groups), especially related to patients’ characteristics and patient portal use. These barriers included *lack of eHealth literacy*. This can be due to the diversity of the patient population because it will include immigrants, older patients, and people with limited literacy skills. These specific groups may experience difficulty using a portal. Patients might also fear using the portal or simply need time to get used to it:

We have a lot of patients with low levels of literacy [...] So a lot of people without digital access to information, and no computer. That is a barrier for the portal in this hospital.

Manager, teaching hospital

#### Social Context

##### Barrier and Facilitator

*Negative attitude or opinion of medical professionals* was seen as a barrier and a facilitator by all stakeholder groups. They stated that this is because of doctors’ resistance regarding transparency of medical information, negative outcome expectancy because they think they will receive more questions and phone calls, and they are sometimes afraid to lose control:

...a lot of professionals are very tense about it. They are used to have the control when they get in touch with a patient or have an appointment with a patient. Now it is possible for patients to interfere with this. Doctors and other professionals are tense about that. So that is a barrier for implementation.

Manager, university medical center

However, *positive attitude or opinion of medical professionals* was seen as a facilitator*.* When medical professionals are enthusiastic, it can facilitate the implementation, and they can influence others in a positive way. It was also mentioned that medical professionals asked for IT services for patients to be improved:

There is also an explicit request from the medical staff to support, what they call patient IT, so that is positive.

IT employee, general hospital

#### Organizational Context

##### Barriers

*Lack of resources*, *lack of time* and *increased workload*, *innovation-averse hospital culture*, *lack of suitable specialist staff*, *difficult to adjust organization of care processes*, *structures of the organization*, and *change in task and new responsibilities* were identified as barriers. *Lack of resources* was seen as a barrier, and although material resources—such as a lack of advanced IT materials—can be a reason, mainly the lack of human resources was mentioned by stakeholders. These resources are not only essential for implementation but also to maintain the portal and to ensure the continuity of service to patients, once the portal has been implemented. IT employees are especially important because this process requires specific knowledge. This technical knowledge is often lacking in hospitals, and it may therefore be necessary to hire suitable specialist staff. This means that there should be enough money to attract resources, which can be a problem because the budgets of hospitals are limited:

An organization has limited resources nowadays, so yes that is a barrier. It is not that we can open a cash box and say we will hire 20 more people to finish this together. That is not how it works.

Medical professional, teaching hospital

The *innovation-averse culture* in hospitals is often identified as a barrier. One reason for this is that each person wants to give his or her opinion (about the portal), and that all opinions need to be taken into account, which inevitably slows down the implementation. Health care is also seen as essentially conservative—especially by managers—meaning that health care organizations and professionals need to get used to a new medium such as a patient portal.

These new services may affect hospitals’ care processes, which can be difficult to adjust. Patients usually have access to their portal 24 hours a day, 7 days a week. If they experience a problem or they ask a question, it should be addressed quickly, and this may not always be possible. *Adjusting the organization of care processes* might be necessary, for example, concerning the transparency of medical information on the portal. Adjusting these care processes can be a barrier because they are sometimes ambiguous and usually difficult to change. This may also lead to *changes in tasks and new responsibilities* for the staff. New tasks or changes in existing work processes and responsibilities may result in informing patients about the portal and answering questions that arise when reading medical information on the portal. But also *lack of time and increased workload* was noted as a barrier, and the time investment required from medical professionals was especially seen as a problem. Furthermore, *organizational structures* can also hinder implementation for the reason that each division in a hospital tends to have its own management, policy agreements, and prioritizing approach.

##### Facilitators

*Management support, communication to promote the portal,* and *innovation-oriented culture* were seen as facilitators. The support of hospitals’ management can facilitate portal implementation, especially when there is a hospital-wide strategy on eHealth—and patient portals—available. On the other hand, if this is missing, then that can be a barrier to implementation. *Management support* and approval can also be a facilitator; it can help the organization to focus on the implementation instead of on the internal discussion whether or not to implement the portal:

...the decision of the board means everything, because then you are not going to discuss if we are going to do it and why but we are going to do this and how [...] that is an absolute must and facilitator for this kind of project to be implemented.

IT employee, university medical center

Clear *communication (to promote the portal)* was indicated to be facilitating and relevant for staff because it can reduce professionals’ misunderstanding, for example, regarding functionalities on the portal. Sessions to inspire staff about eHealth can facilitate implementation, and hospitals can use publicity to raise awareness about the availability of the portal and thereby increase accessibility for patients.

An *innovation-oriented culture* can help for the reason that the implementation is supported by the organization, the staff are stimulated and feel motivated, and there is a positive mood.

#### Economic and Political Context

##### Barrier

*Financial difficulties* were seen as a barrier mainly because funding is often a problem, and technical adjustments are expensive. In addition, the reimbursement for certain applications, for example, e-consults, has not yet been arranged:

The barrier is that it is not directly insured care, it is a bit luxurious (to provide it to patients now). So you have to find funding for it.

Medical professional, general hospital

##### Facilitator

*Facilitating laws and regulations* can be beneficial, and especially the support by the government in the Netherlands for portal implementation is seen as a facilitator.

### Comparison of Stakeholder Groups

We found similarities between stakeholders, for example, regarding *perceived usefulness*, but also differences (Table 4). Overall, the findings regarding *lack of resources* were fairly similar among the groups, although the majority (5/7, 71%) of the IT employees also mentioned that there are *sufficient resources* available. *Guaranteeing privacy and security* was mentioned by both managers (5/7, 71%) and IT employees (5/7, 71%) as a barrier. The majority of medical professionals (4/7, 57%) and managers (5/7, 71%) mentioned *lack of sufficient eHealth literacy* of patients as a barrier.

However, we also found differences between stakeholder groups. The *negative attitude or opinion of medical professionals* was often seen as a barrier, especially by medical professionals. They were most often negative about providing patients with medical information via the patient portal because they were afraid it would lead to more work (such as more questions from patients), and they were worried about losing control. A remarkable finding is that all the managers (7/7, 100%) see their own *positive attitude* as a facilitator; however, this is true for only less than the half (3/7, 43%) of the other groups. All the medical professionals mentioned the *perceived usefulness* of the portal, but they (4/7, 57%) also indicated a *lack of perceived usefulness* because they think that the portal can lead to practical problems. However, the majority of this group is *motivated to change* (4/7, 57%) compared with only a minority in the other 2 stakeholder groups (both 2/7, 29%).

**Table 4:** Top 3 barriers and facilitators for each stakeholder group and ranked by number of subjects.

Barriers and facilitators by stakeholder group		n (%)
**Medical professionals (n=7)**		
	Perceived usefulness (+^a^)	7 (100)
	Financial difficulties (−^b^)	5 (71)
	Lack of perceived usefulness (−)	4 (57)
	Motivation to change (+)	4 (57)
	Lack of sufficient eHealth literacy (−)	4 (57)
	Negative attitude or opinion of medical professionals (−)	4 (57)
	Lack of resources (−)	4 (57)
	Lack of time and increased workload (−)	4 (57)
**Managers (n=7)**		
	Perceived usefulness (+)	7 (100)
	Positive attitude (+)	7 (100)
	Financial difficulties (−)	6 (86)
	Guaranteeing privacy and security (−)	5 (71)
	Lack of sufficient eHealth literacy (−)	5 (71)
	Lack of resources (−)	5 (71)
**IT^c^ employees (n=7)**		
	Perceived usefulness (+)	7 (100)
	Lack of resources (−)	6 (86)
	Guaranteeing privacy and security (−)	5 (71)
	Sufficient resources (+)	5 (71)

^a^“+” indicates facilitator.

^b^“–” indicates barrier.

^c^IT: information technology.

### Comparison of Hospital Types

In Table 5, the top 3 barriers and facilitators for each hospital type are listed. A complete overview of all barriers and facilitators—including the number of subjects for each hospital type—is presented in Multimedia Appendix 4. Differences were found in the barriers mentioned by subjects from different hospital types. The majority (5/6, 80%) of subjects from UMCs mentioned *lack of accessibility* as a barrier, and the difficult login method was especially seen as a barrier in these hospitals. In general hospitals, most subjects think that the *positive attitude or opinion of medical professionals* will facilitate implementation because medical professionals are enthusiastic. *Lack of time and increased workload* is also an important barrier in general hospitals because everybody is already always busy. Along with the differences, we also found similarities between the 3 hospital types. *Perceived usefulness* was mentioned by all subjects (21/21, 100%), but also *lack of resources* was seen in every hospital type as an important barrier. The UMCs and general hospitals see that the *lack of sufficient eHealth literacy* can hinder patient portal use. The most similarities were found between the teaching and general hospitals. *Positive attitude*, *guaranteeing privacy and security,* and *financial difficulties* were mentioned by the majority of subjects in both teaching and general hospitals. This is an important difference from the UMCs, which can perhaps be explained by differences in the financing of these hospital types.

**Table 5:** Barriers and facilitators—top 3 for each hospital type and ranked by number of subjects.

Barriers and facilitators by hospital type		n (%)
**UMCs^a^ (n=6)**		
	Perceived usefulness (+^b^)	6 (100)
	Lack of accessibility (−^c^)	5 (83)
	Lack of sufficient eHealth literacy (−)	4 (67)
	Lack of resources (−)	4 (67)
**Teaching hospitals (n=9)**		
	Perceived usefulness (+)	9 (100)
	Lack of resources (−)	7 (78)
	Financial difficulties (−)	7 (78)
	Guaranteeing privacy and security (−)	6 (67)
	Positive attitude (+)	6 (67)
**General hospitals (n=6)**		
	Perceived usefulness (+)	6 (100)
	Positive attitude (+)	5 (83)
	Guaranteeing privacy and security (–)	4 (67)
	Lack of sufficient eHealth literacy (–)	4 (67)
	Positive attitude or opinion of medical professionals (+)	4 (67)
	Lack of resources (–)	4 (67)
	Lack of time and increased workload (–)	4 (67)
	Financial difficulties (–)	4 (67)

^a^UMC: university medical center.

^b^“+” indicates facilitator.

^c^“−” indicates barrier.

### Comparison of Hospitals With and Without an Implemented Patient Portal

Although we did not explicitly ask the included hospitals in which phase of implementation they were, we could deduce this from the interviews. In total, we included 7 hospitals. Two of these hospitals had no patient portal but were planning implementation. Three hospitals had minimal experience with portals—small pilots with limited functionalities or a classic portal version—but were also in the implementation phase. Only 2 hospitals had an active patient portal; however, stakeholders of one hospital mentioned they were still implementing to extend their current functionalities. In Table 6, we list the barriers and facilitators that were mentioned by (at least one stakeholder) all the included hospitals both with a patient portal (n=2) and without a patient portal (n=5). A complete overview is presented in Multimedia Appendix 5. Although there were similarities (eg, *financial difficulties, lack of sufficient eHealth literacy)*, we also found differences. All hospitals without a patient portal mentioned *negative attitude or opinion of medical professionals* and *lack of specialist staff* as barriers. These factors could negatively influence implementation. Although the hospitals with a patient portal see barriers for the implementation of their patient portals, they also mentioned multiple facilitators, for example, *perceived ease of use, motivation to change,* and *sufficient resources*. The barriers *lack of a generic guideline* (n=1) and *participation of end users during implementation* (n=1) were only mentioned by hospitals with a patient portal. *Lack of a generic guideline* was a barrier expressed by a manager (n=1), meaning that it could have been beneficial for implementation if there would have been coordination or a standard format. All stakeholders of one hospital that had implemented a portal noticed *participation of end users during implementation.* In that case, they referred back to the implementation and stated that it was useful to involve end users—both patients and health care professionals—during implementation and for each hospital division to be well represented in the project organization.

**Table 6:** Barriers and facilitators mentioned by all hospitals (at least one subject per hospital) with and without a patient portal and ranked by total number of subjects.

Barriers to and facilitators of hospitals with and without a patient portal			Hospitals with a patient portal^a^, n (%)	Hospitals without a patient portal^b^, n (%)
**Barriers and facilitators common for hospitals with and without a patient portal (ie, unanimously reported by hospitals of both groups)**				
	**Barriers**			
		Financial difficulties	4 (67)	10 (67)
		Lack of sufficient eHealth literacy	4 (67)	9 (60)
		Lack of resources	2 (33)	12 (80)
		Negative attitude or opinion of colleagues in general	3 (50)	9 (60)
	**Facilitators**			
		Perceived usefulness	6 (100)	15 (100)
		Positive attitude	3 (50)	10 (67)
**Barriers and facilitators only reported unanimously by hospitals with a patient portal**				
	**Barriers**			
		Lack of time and increased workload	4 (67)	
		Innovation-averse culture	3 (50)	
		Adjusting organization of care processes	3 (50)	
		Structures of the organization	3 (50)	
		Change in task and new responsibilities	2 (33)	
	**Facilitators**			
		Perceived ease of use	3 (50)	
		Motivation to change	2 (33)	
		Having knowledge	2 (33)	
		Positive attitude or opinion of medical professionals	2 (33)	
		Good collaboration with colleagues	2 (33)	
		Sufficient resources	2 (33)	
		Conducive financial arrangements	2 (33)	
**Barriers only reported unanimously by hospitals without a patient portal**				
		Negative attitude or opinion of medical professionals		7 (47)
		Lack of suitable specialist staff		5 (33)

^a^n=2 hospitals; n=6 subjects.

^b^n=5 hospitals; n=15 subjects.

## Discussion

### Summary of Main Findings

In this study, we have presented an overview of the barriers and facilitators related to patient portal implementation among various stakeholders within the hospital organization. In total, we identified 26 barriers and 28 facilitators. Positive factors related to *perceived usefulness* (eg, cost savings, accessibility for patients to their information) were mentioned by all subjects. The facilitators individuals’ *positive attitude* and *management support* (eg, strategy plan for eHealth and patient portals) were also mentioned by majority of the subjects. The main barriers reported were *lack of resources* (especially lack of staff), *financial difficulties* (high costs, lack of reimbursement), and *guaranteeing privacy and security* (eg, strict regulations). We want to emphasize that no inferences can be drawn about the prevalence of phenomena observed beyond the current sample.

We found several similarities between stakeholders (eg, regarding *perceived usefulness*) but also remarkable differences that highlight the importance of involving multiple stakeholders. One interesting finding is that approximately half the medical professionals see their own *positive attitude* and *motivation to change* as facilitators. Although medical professionals’ motivation to change is the highest of all stakeholder groups, *lack of time and increased workload* was perceived by them as a barrier. Apparently, they are willing to change, but at the same time, they assume that they do not have enough time to achieve implementation and portal use. The barriers *guaranteeing privacy and security* and *lack of resources* were mentioned by the majority of IT employees. This shows the challenges this group is dealing with when implementing a secure portal. Managers were the only group of which all (7/7, 100%) stated that they had a *positive attitude.* This is in clear contrast with the proportion of medical professionals and IT employees (both 3/7, 43%). Managers also stand out in their statements about the culture with more than the half of the managers (4/7, 57%) thinking the culture is hindering implementation, whereas only a minority of both the medical professionals (1/7, 14%) and IT employees (1/7, 14%) stated this. Managers mentioned that hospital culture is conservative and slow to change.

### Comparison With Previous Research

Koivunen et al [28] identified nurses’ barriers and facilitators regarding portal implementation. Their findings were comparable with ours; for example, concerning the barriers *lack of resources* and *lack of time*. However, in their study, nurses were included and were mainly negative because they had doubts about the benefits of the portal; moreover, they were unwilling to use a new technical tool because they believed that their primary tasks are to be more important. This differs from our findings as we found *positive attitudes* among all included stakeholders (medical professionals, managers, and IT employees), and all our subjects mentioned *perceived usefulness* as a facilitator for patient portal implementation. One reason for these differences may be the selection of stakeholders, as we focused on those directly involved and did not include nurses, only medical doctors and nurse practitioners (“Advanced Practice Registered Nurses”) [30]. Keplinger et al [27] also considered physicians’ attitudes regarding patient portal implementation. Some of their findings are in line with ours, for example, the expected increase in workload and positive attitudes regarding the patient portal. However, they also found differences in attitudes both before and after implementation. For example, before implementation, more than half of the physicians assumed that their workload would increase, whereas only one-third actually experienced such an increase in workload.

McGinn et al [21] showed the relevance of including the perspectives of various stakeholders regarding EHR implementation. Their results are both similar and different from our results. They found that the main factors common to all stakeholder groups were found at various levels and included “perceived ease of use,” “costs,” “motivation to use EHR,” and “privacy and security concerns.” These findings are similar to ours perhaps because *financial difficulties, guaranteeing privacy and security,* and *positive attitude* were mentioned by the majority of our subjects. The use of the internet and other electronic applications is becoming increasingly common in health care [33], and patients’ eHealth literacy needs to be taken into account. *Participation of end users during implementation* was mentioned as a facilitator and can be used to focus on the eHealth literacy of the users.

McGinn et al [21] argue that the consideration of various stakeholder opinions may contribute to successful EHR implementations. Similarities with and differences from our results were found. The main factors common to all stakeholder groups were found at various levels and included “design and technical concerns,” “costs,” “lack of time and workload,” and “privacy and security.” The findings are similar to ours, and this can be the case because both EHRs as well as patient portals are complex technologies that affect multiple levels of an organization. However, we also found differences because in our study, *perceived usefulness* and *lack of sufficient eHealth literacy (patients)* were mentioned by the majority of the subjects. *Lack of accessibility* (because of login methods perceived as difficult) was mentioned by almost half of the subjects. This difference can be due to an EHR being primarily aimed at professionals and a patient portal being primarily intended as a service for patients. The differences found among these implementation studies highlight the importance of identifying barriers and facilitators for each technology separately taking into account the perspectives of the several stakeholder groups that are involved.

### Implementation Frameworks and Models

There are many implementation models, and they have considerable overlap [34]. A combination of 2 models was used for categorization of the selected quotes, that is, the model of Grol and Wensing [26] for socio-dynamic factors and by McGinn [21] mainly for portal characteristic and technical factors. Although this combination of frameworks appeared to be a feasible approach, we also added categories and renamed existing ones, so they better match with our findings. An essential difference between our approach and, the CFIR framework is that in our study, patients are included as a separate factor, whereas in the CFIR framework, they are part of the “outer setting” [24]. In the FITT framework, separate categories such as “social context” and “organizational context” are missing, and the aspects related to social interaction, for example, are categorized under “individual” within the FITT model. We found these categories to be relevant as a separate level because many subjects reported on them [25]. In the McGinn model [21], a subcategory is “participation of end users during the design,” which does not cover all the input we received, particularly because it is not aimed at the complete implementation process. One of the added categories is *participation of end users during implementation*. Another new category is *sufficient eHealth literacy,* which encompasses the skills and knowledge necessary to use electronic applications [33]. The models we used only address patients’ skills and knowledge [26] and applicability—of EHR implementation—to patients’ characteristics [21]. Patients’ lack of eHealth literacy was identified as a barrier by the majority of the subjects.

### Practical Suggestions and Insights for Portal Implementations

Our findings suggest that implementation is affected by barriers and facilitators at various levels. McGinn et al [21] describe 3 key levels: the macro, meso, and micro levels. We present some suggestions and insights for organizations that intend to implement a patient portal.

#### Micro Level: Individual and Social Factors

Our findings suggest that stakeholders’ *positive attitudes* can contribute to implementation. They greatly value their colleagues’ opinions, so apparently this can play a crucial role in the implementation process. Clear communication with all stakeholders during the implementation process and about the patient portal functionalities can increase stakeholders’ understanding and can help to avoid misunderstandings.

#### Meso Level: Organizational and Operational Developments

The implementation can be affected by operational factors in the organization [21]; for example, *lack of resources*, *management support*, and *lack of suitable specialist staff*. To successfully implement a patient portal, a project team is essential that includes resources and staff with technical knowledge about patient portals and implementation processes. Management support is important; for example, by including the plan for portal implementation in their organizational strategy. Organizations should also be aware that the implementation of a patient portal is not only a technical implementation but also involves a change in the organizational socio-dynamics, including changes in employees’ tasks, new responsibilities, and a shift in control from health care professionals to patients.

#### Macro Level: Sociopolitical Influences

Governments in Western countries are increasingly promoting and supporting portal implementation and use. In the United States, financial support is generated by the Health Information Technology for Economic and Clinical Health Act and arranged by the Centers for Medicare and Medicaid Services. The goal of these incentive programs is to support the implementation [35], adoption, and “meaningful use” of the EHRs [6,35,36]. This includes, for example, providing patients with access to or acquiring an electronic copy of their health data [36]. In the Netherlands, the Ministry of Health and the Dutch Hospital Association developed a funding program to support information exchange for both patients and professionals. The ultimate goal of this program is that in 2020, all Dutch people will have access to their own medical information. Therefore, all institutions must have a patient portal by the end of 2019 or a link to a Personal Health Record (PHR) to which the institution can upload medical information [37]. Government commitment thus can be beneficial for hospitals, especially in view of the opportunities for funding. Hospitals can exploit governments’ ambitions and policies and patient representatives demands, for example, to make EHR data accessible for every patient, as a motivation to facilitate implementation.

### Limitations

This study has several limitations. First, we used semistructured interviews in which we provided participants with prompts/examples for each level. Providing subjects with examples may have restricted participants in their answers about new barriers and facilitators or to “think outside the box” on these topics, so we might have missed factors. However, we used the combined models of Grol and Wensing [26] and McGinn et al [21], and many stakeholders mentioned barriers and facilitators that fell outside our scope. Although we have confidence in the richness of the current data, we already reached data saturation after 6 hospitals, limiting the total number of hospitals and subjects. There were also differences in the included hospitals with regard to the phase of patient portal implementation. Some had already provided a portal, whereas others were in the middle of the implementation process or had no portal at all. Although we found only limited differences between the hospitals with and without an implemented patient portal, this could still have introduced bias into the responses because of the recall or the imagination of information. This means that the results might have been influenced by the current state of hospitals because participants sometimes had to recall information from the time of implementation or had to imagine an implementation process (if there is no portal or no implementation).

Although we presented many different types of barriers and facilitators, we acknowledge that quantity should not be taken as a proxy for importance. We therefore added quotes to the results so as to highlight the specific nature of specific barriers and facilitators. For data analysis, we used a directed content analysis (deductive) approach. This can be a possible limitation because we started with an already existing model with defined categories. However, as the methods allows, we did not completely hold on to the categories in the models as we added additional categories ourselves and renamed the existing (generic) categories to barriers and facilitators that better fit our findings. Despite these limitations this is, to the best of our knowledge, the first qualitative study to identify barriers and facilitators for patient portal implementation involving multiple stakeholder groups.

### Future Perspectives and Research Directions

Instead of organizing health care around professionals and institutions, some contend that it should increasingly be arranged around patients [2]. In a recent review, we found little evidence for the efficacy of IT-supported shared care [38]; however, many initiatives exist that may facilitate patient-centered or shared care. We already see movement in this direction as information systems are evolving from purely organizational to regional and even international systems [39]. For instance, a PHR is an example of an application in which patients can access their health information that has been collected from various health care institutions but is controlled by the patients [40]. In several European countries, these national systems have already been introduced. For example, in France, there is a national initiative called “Dossier Médical Personnel,” which is accessible over the internet. The information is uploaded by the involved clinicians; however, patients are in charge about what is included in the portal and who is authorized to access it. In Estonia, health professionals transfer information into a system called the “Estonian Health Information System,” providing patients with information via a patient portal [41]. These initiatives show a shift from hospital-financed, -owned, and -managed health records for which access is granted through portals, toward PHRs in which providers upload the data and ownership by patients is facilitated. The present uptake/compliance rates of portals are however still rather low (seldom above 50%), so this is an aspect that should receive attention if widespread use is foreseen.

Future research is necessary to confirm the practical utility of our proposed model when used among various stakeholder groups and to test whether it is useful to tailor implementation strategies to these various stakeholders, and organizations, taking possible development routes into account. In addition, there is a lack of knowledge regarding the association between patient portal implementation and patient portal adoption (ie, actual uptake and use by patients). One important element we identified is eHealth literacy, and this should ideally be included in the implementation and evaluation strategies for health technology tools. Moreover, the expectations before implementations and the experiences afterward can vary among health care professionals [27] and patients [11]. Further research into “satisfiers” determining the attitude of professionals toward using these technologies is recommended because evidence of the effectiveness of technology-related aspects on patient empowerment and on health outcomes is a strong facilitator.

### Conclusions

Patient portal implementation is a complex process that is not just a technical process, but it also affects an organization and its staff. We found barriers and facilitators at various levels that differed depending on hospital types (eg, *lack of accessibility*) and stakeholder groups (eg, *sufficient resources*) in terms of several factors. Our findings underscore the importance of involving multiple stakeholders in portal implementation projects. We identified a set of barriers and facilitators, which are likely to be useful in making strategic and efficient portal implementation plans.

## Appendix

Multimedia Appendix 1 Interview questions.

Multimedia Appendix 2 Barriers and facilitators categorized according to the model of Grol & Wensing and the model of McGinn et al.

Multimedia Appendix 3 Barriers and facilitators for each stakeholder group.

Multimedia Appendix 4 Barriers and facilitators for each hospital type.

Multimedia Appendix 5 Barriers and facilitators for hospitals with and without a patient portal.

## Abbreviations

CFIR: Consolidated Framework for Implementation Research
EHR: electronic health record
FITT: Fit between Individuals, Tasks, and Technology
IT: information technology
PHR: personal health record
UMC: university medical centers
