# Supplementary material for: Barriers and Facilitators Affecting Patient Portal Implementation from an Organizational Perspective: Qualitative Study
Source: J Med Internet Res. 2018 May 11;20(5):e183. doi: 10.2196/jmir.8989 (PMC5970285; doi:10.2196/jmir.8989)
Supplement: Multimedia Appendix 1 [file jmir_v20i5e183_app1.pdf]

Multimedia Appendix 1: Interview questions.

Interview Questions

1. Participants were first asked for their consent to make audio recordings of the interviews. After that, questions were asked about participants' characteristics, such as their age and work experience. We also asked participants what their definition of a patient portal was, and if necessary, it was complemented with our definition. Our definition: "a patient portal is a personal digital environment, facilitated by a health care institution, for example a hospital. Patients need to login to the portal to get access to, for example, their medical file (with results), patient information and appointments. Patients can also fill in questionnaires and receive personalized advice regarding, for example, quality of life and physical activity."

2. Barriers and facilitators

A. Individual professional.

Do you, as a individual professional, anticipate barriers to and facilitators for implementing a patient portal?

If yes, which barriers and facilitators?

Examples:

- Your knowledge regarding the implementation of a patient portal
- Your attitude regarding the implementation of a patient portal
- Your motivation regarding the implementation of a patient portal

B. Patient

Do you anticipate barriers and/or facilitators for patients using a patient portal?

If yes, which barriers/facilitators?

Examples:

- Patients' knowledge about a patient portal
- Patients' skills in using a patient portal
- Patients' attitude regarding a patient portal

C. Social context

Do you anticipate barriers and/or facilitators (regarding the implementation of a patient portal) concerning the social context in your organization?

Examples:

- Opinion of colleagues
- Culture within the organization
- Collaboration

D. Organizational context

Do you anticipate barriers and/or facilitators (regarding the implementation of a patient portal) concerning the social context in your organization?

Examples:

- Organization of care processes
- Staff
- Resources

E. Economic and political context

Do you anticipate barriers and/or facilitators (regarding the implementation of a patient portal) concerning the social context in your organization?

Examples:

- Financial arrangements
- Laws and regulations
- Policy

F. Patient portal characteristics

Do you anticipate barriers and/or facilitators (regarding the implementation of a patient portal) concerning the patient portal characteristics?

Examples:

- Accessibility of the patient portal
- Attractiveness of the patient portal
- Ease of use of the patient portal
- Credibility of the content of the patient portal
