# Supplementary material for: Barriers and Facilitators Affecting Patient Portal Implementation from an Organizational Perspective: Qualitative Study
Source: J Med Internet Res. 2018 May 11;20(5):e183. doi: 10.2196/jmir.8989 (PMC5970285; doi:10.2196/jmir.8989)
Supplement: Multimedia Appendix 2 [file jmir_v20i5e183_app2.pdf]

Multimedia Appendix 2: Barriers and facilitators categorized according to the model of Grol & Wensing and the model of McGinn.

Barriers and facilitators	Grol & Wensing [26]	McGinn [21]
**Innovation**		
Barriers/facilitators		
Guaranteeing privacy and security/Privacy and security		Privacy and security concerns
Lack of accessibility/Good accessibility	Accessibility	
Lack of attractiveness/Attractiveness	Attractiveness	
Lack of interoperability/Interoperability with EHR		Interoperability
Lack of perceived usefulness/Perceived usefulness		Perceived usefulness
Lack of tailored content/Content tailored to patients		Content appropriate for the users (relevance)
Facilitators		
Credibility	Credibility	
Participation of end-users during implementation		Participation of end-users in the design
Perceived ease of use		Perceived ease of use
Perceived usefulness		Perceived usefulness
**Individual professional**		
Barriers/facilitators		
Lack of knowledge/Having knowledge	Knowledge	Knowledge (main category)
Lack of motivation to change/Motivation to change	Motivation to change	Motivation/inertia to use EHR (readiness)/resistance to use the EHR
Facilitator		
Positive attitude	Attitude	Attitude (main category)
**Patient**		
Barriers/facilitators		
Lack of sufficient eHealth literacy/Sufficient eHealth literacy		
Negative attitude/lack of need/Positive		Patients' attitudes and

attitude/demand		preferences towards EHR
**Social Context**		
Barriers/facilitators		
Negative attitude or opinion of colleagues in general/Positive attitude and opinion of colleagues in general	Opinion of colleagues	Attitude of colleagues about EHR
Negative attitude or opinion of medical professionals/Positive attitude or opinion of medical professionals	Opinion of colleagues	Attitude of colleagues about EHR
Barrier		
Varying opinions about IT security	Opinion of colleagues	
Facilitators		
Varying opinions about implementation	Opinion of colleagues	
Early adapters		
Good collaboration with colleagues	Collaboration	
**Organizational context**		
Barriers/facilitators		
Lack of suitable specialist staff/suitable staff	Staff	
Lack of resources/sufficient resources	Resources	Resources available/Material resources (access to EHR)/Human resources (IT support, other)
Innovation-averse hospital culture/Innovation oriented-hospital culture	Culture of the networks (social context)	Innovation culture
Barriers		
Adjusting organization of care processes is difficult	Organization of care process	
Change in task and new responsibilities		Change in task
Lack of time and increased workload		Lack of time and workload
No strategic plan and lack of organizational priority/management support		Management (strategic plan to implement EHR)
Organization is not ready for implementation	Readiness	
Structure of the organization	Structures	
Facilitator		
Communication to promote the portal		Communication (included promotional activities)
**Economic and political context**		

Barriers/facilitators		
Financial difficulties/conducive financial arrangements	Financial arrangements	Financing of EHR/Financial support/Cost issues
Third-party dependency/Good collaboration with third parties		
Lack of generic guidelines	Policies	
Restrictions imposed by laws and regulations/ Facilitating law- and regulations	Regulations	
Supporting healthcare policies	Policies	Health care policies and socio political context
