# Supplementary material for: Barriers and Facilitators Affecting Patient Portal Implementation from an Organizational Perspective: Qualitative Study
Source: J Med Internet Res. 2018 May 11;20(5):e183. doi: 10.2196/jmir.8989 (PMC5970285; doi:10.2196/jmir.8989)
Supplement: Multimedia Appendix 3 [file jmir_v20i5e183_app3.pdf]

Multimedia Appendix 3: Barriers to and facilitators of patient portal implementation for each stakeholder group and ranked by number of subjects.

Barrier and facilitators	Stakeholders			Total (n=21), n (%)
	Medical professionals[a], n (%)	Managers[a], n (%)	IT[b] employees[a], n (%)	
**Innovation: patient portal**				
**Barriers**				
Guaranteeing privacy and security	1 (14)	5 (71)	5 (71)	11 (52)
Lack of accessibility	2 (29)	4 (57)	3 (43)	9 (43)
Lack of perceived usefulness	4 (57)	1 (14)	2 (29)	7 (33)
Lack of interoperability	0 (0)	1 (14)	1 (14)	2 (10)
Lack of attractiveness	0 (0)	1 (14)	0 (0)	1 (5)
Lack of tailored content	1 (14)	0 (0)	0 (0)	1 (5)
**Facilitators**				
Perceived usefulness	7 (100)	7 (100)	7 (100)	21 (100)
Perceived ease of use	2 (29)	2 (29)	1 (14)	5 (24)
Attractiveness	1 (14)	1 (14)	2 (29)	4 (19)
Participation of end users during implementation	1 (14)	1 (14)	1 (14)	3 (14)
Privacy and security	2 (29)	0 (0)	1 (14)	3 (43)
Good accessibility	0 (0)	2 (29)	0 (0)	2 (10)
Credibility	0 (0)	2 (29)	0 (0)	2 (10)
Content tailored to patients	0 (0)	1 (14)	0 (0)	1 (5)
Interoperability with EHR	0 (0)	0 (0)	1 (14)	1 (5)
**Individual professional**				

**Barriers**				
Lack of knowledge	0 (0)	2 (29)	2 (29)	4 (19)
Lack of motivation to change	1 (14)	0 (0)	0 (0)	1 (5)
**Facilitators**				
Positive attitude	3 (43)	7 (100)	3 (43)	13 (62)
Motivation to change	4 (57)	2 (29)	2 (29)	8 (38)
Having knowledge	1 (14)	2 (29)	2 (29)	5 (24)
**Patient**				
**Barriers**				
Lack of sufficient eHealth literacy	4 (57)	5 (71)	4 (57)	13 (62)
Negative attitude/lack of need	0 (0)	2 (29)	0 (0)	2 (10)
**Facilitators**				
Sufficient eHealth literacy	2 (29)	2 (29)	0 (0)	4 (19)
Positive attitude/demand	1 (14)	0 (0)	1 (14)	2 (10)
**Social context**				
**Barriers**				
Negative attitude or opinion of medical professionals	4 (57)	3 (43)	1 (14)	8 (38)
Negative attitude or opinion of colleagues in general	3 (43)	0 (0)	3 (43)	6 (29)
Varying opinions about IT security	0 (0)	0 (0)	1 (14)	1 (5)
**Facilitators**				
Positive attitude or opinion of colleagues in general	0 (0)	2 (29)	4 (57)	6 (29)
Positive attitude or opinion of medical professionals	1 (14)	2 (29)	2 (29)	5 (24)
Good collaboration with colleagues	0 (0)	2 (29)	2 (29)	4 (19)
Early adopters	0 (0)	3 (43)	0 (0)	3 (14)
Varying opinions about implementation	1 (14)	0 (0)	0 (0)	1 (5)

**Organizational context**				
**Barriers**				
Lack of resources	4 (57)	5 (71)	6 (86)	15 (71)
Lack of time and increased workload	4 (57)	3 (43)	1 (14)	8 (38)
Innovation-averse culture	1 (14)	4 (57)	1 (14)	6 (29)
Lack of suitable specialist staff	1 (14)	2 (29)	3 (43)	6 (29)
Adjusting organization of care processes is difficult	2 (29)	1 (14)	2 (29)	5 (24)
Structure of the organization	2 (29)	1 (14)	2 (29)	5 (24)
Change in task and new responsibilities	1 (14)	1 (14)	2 (29)	4 (19)
Organization is not ready for implementation	2 (29)	2 (29)	0 (0)	4 (19)
No strategic plan and lack of organizational priority	0 (0)	2 (29)	0 (0)	2 (10)
**Facilitators**				
Management support	2 (29)	3 (43)	3 (43)	8 (38)
Communication to promote the portal	1 (14)	4 (57)	1 (14)	6 (29)
Sufficient resources	1 (14)	0 (0)	5 (71)	6 (29)
Innovation-oriented culture	2 (29)	2 (29)	1 (14)	5 (24)
Suitable specialist staff	0 (0)	1 (14)	0 (0)	1 (5)
**Economic and political context**				
**Barriers**				
Financial difficulties	5 (71)	6 (86)	3 (43)	14 (67)
Restrictions imposed by laws and regulations	0 (0)	3 (43)	1 (14)	4 (19)
Third-party dependency	0 (0)	1 (14)	1 (14)	2 (10)
Lack of generic guidelines	0 (0)	1 (14)	0 (0)	1 (5)
**Facilitators**				
Facilitating laws and regulations	1 (14)	2 (29)	1 (14)	4 (19)

Conducive financial arrangements	0 (0)	2 (29)	1 (14)	3 (14)
Good collaboration with third parties	0 (0)	1 (14)	1 (14)	3 (14)
Supporting healthcare policies	0 (0)	3 (43)	0 (0)	3 (14)

[a]n=7

[b]IT: information technology
