# Supplementary material for: Barriers and Facilitators Affecting Patient Portal Implementation from an Organizational Perspective: Qualitative Study
Source: J Med Internet Res. 2018 May 11;20(5):e183. doi: 10.2196/jmir.8989 (PMC5970285; doi:10.2196/jmir.8989)
Supplement: Multimedia Appendix 4 [file jmir_v20i5e183_app4.pdf]

Multimedia Appendix 4: Barriers to and facilitators of patient portal implementation for each hospital type and ranked by number of subjects.

Barriers and facilitators	Hospital types			Total (n=21), n (%)
	UMC[a], n (%)	Teaching hospitals[b], n (%)	General hospitals[a], n (%)	
**Innovation: patient portal**				
**Barriers**				
Guaranteeing privacy and security	1 (17)	6 (67)	4 (67)	11 (52)
Lack of accessibility	5 (83)	4 (44)	0 (0)	9 (43)
Lack of perceived usefulness	1 (17)	4 (44)	2 (33)	7 (33)
Lack of interoperability	0 (0)	2 (22)	0 (0)	2 (10)
Lack of attractiveness	0 (0)	1 (11)	0 (0)	1 (5)
Lack of tailored content	0 (0)	1 (11)	0 (0)	1 (5)
**Facilitators**				
Perceived usefulness	6 (100)	9 (100)	6 (100)	21 (100)
Perceived ease of use	2 (33)	2 (22)	1 (17)	5 (24)
Attractiveness	0 (0)	2 (22)	2 (33)	4 (19)
Participation of end-users during implementation	3 (50)	0 (0)	0 (0)	3 (14)
Privacy and security	1 (17)	2 (22)	0 (0)	3 (14)
Good accessibility	0 (0)	1 (11)	1 (17)	2 (10)
Credibility	1 (17)	1 (11)	0 (0)	2 (10)
Content tailored to patients	0 (0)	1 (11)	0 (0)	1 (5)
Interoperability with EHR	0 (0)	0 (0)	1 (17)	1 (5)
**Individual professional**				
**Barriers**				
Lack of knowledge	2 (33)	1 (11)	1 (17)	4 (19)

Lack of motivation to change	0 (0)	0 (0)	1 (17)	1 (5)
**Facilitators**				
Positive attitude	2 (33)	6 (67)	5 (83)	13 (62)
Motivation to change	3 (50)	3 (33)	2 (33)	8 (38)
Having knowledge	1 (17)	3 (33)	1 (17)	5 (24)
**Patient**				
**Barriers**				
Lack of sufficient eHealth literacy	4 (67)	5 (55)	4 (67)	13 (62)
Negative attitude/lack of need	1 (17)	1 (11)	2 (33)	4 (19)
**Facilitators**				
Sufficient eHealth literacy	0 (0)	2 (22)	2 (33)	4 (19)
Positive attitude/demand	1 (17)	1 (11)	0 (0)	2 (10)
**Social context**				
**Barriers**				
Negative attitude or opinion of medical professionals	3 (50)	4 (44)	1 (17)	8 (38)
Negative attitude or opinion of colleagues in general	3 (50)	2 (22)	1 (17)	6 (29)
Varying opinions about IT security	0 (0)	0 (0)	1 (17)	1 (5)
**Facilitators**				
Positive attitude or opinion of colleagues in general	3 (50)	3 (33)	0 (0)	6 (29)
Positive attitude or opinion of medical professionals	1 (17)	0 (0)	4 (67)	5 (24)
Good collaboration with colleagues	2 (33)	0 (0)	2 (33)	4 (19)
Early adopters	1 (17)	1 (11)	1 (17)	3 (14)
Varying opinions about implementation	0 (0)	1 (11)	0 (0)	1 (5)
**Organizational context**				
**Barriers**				

Lack of resources	4 (67)	7 (78)	4 (67)	15 (71)
Lack of time and increased workload	2 (33)	2 (22)	4 (67)	8 (38)
Innovation-averse hospital culture	2 (33)	3 (33)	1 (17)	6 (29)
Lack of suitable specialist staff	2 (33)	3 (33)	1 (17)	6 (29)
Adjusting organization of care processes is difficult	3 (50)	1 (11)	1 (17)	5 (24)
Structure of the organization	1 (17)	2 (22)	2 (33)	5 (24)
Change in task and new responsibilities	3 (50)	0 (0)	1 (17)	4 (19)
Organization is not ready for implementation	1 (17)	0 (0)	2 (33)	3 (14)
No strategic plan and lack of organizational priority	1 (17)	0 (0)	1 (17)	2 (10)
**Facilitators**				
Management support	3 (50)	5 (55)	0 (0)	8 (38)
Communication to promote the portal	2 (33)	3 (33)	1 (17)	6 (29)
Sufficient resources	2 (33)	2 (22)	1 (17)	5 (24)
Innovation-oriented culture	0 (0)	4 (44)	1 (17)	5 (24)
Suitable specialist staff	0 (0)	0 (0)	1 (17)	1 (5)
**Economic and political context**				
**Barriers**				
Financial difficulties	3 (50)	7 (78)	4 (67)	14 (67)
Restrictions imposed by laws and regulations	2 (33)	1 (11)	1 (17)	4 (19)
Third-party dependency	1 (17)	0 (0)	1 (17)	2 (10)
Lack of generic guidelines	0 (0)	0 (0)	1 (17)	1 (5)
**Facilitators**				
Facilitating law- and regulations	2 (33)	2 (22)	0 (0)	4 (19)
Conducive financial arrangements	1 (17)	1 (11)	1 (17)	3 (14)
Supporting healthcare policies	1 (17)	1 (11)	1 (17)	3 (14)

Good collaboration with third parties	1 (17)	0 (0)	1 (17)	2 (10)

[a] total n=2 hospitals; total n=6 subjects

[b] total n=3 hospitals; total n=9 subjects
