# Supplementary material for: Barriers and Facilitators Affecting Patient Portal Implementation from an Organizational Perspective: Qualitative Study
Source: J Med Internet Res. 2018 May 11;20(5):e183. doi: 10.2196/jmir.8989 (PMC5970285; doi:10.2196/jmir.8989)
Supplement: Multimedia Appendix 5 [file jmir_v20i5e183_app5.pdf]

Multimedia Appendix 5: Barriers to and facilitators of patient portal implementation for hospitals with and without a patient portal and ranked by number of subjects.

Barriers and facilitators	Hospitals with a patient portal[a], n (%)	Hospitals without a patient portal[b], n (%)
**Innovation: patient portal**		
**Barriers**		
Guaranteeing privacy and security	2 (33)	9 (60)
Lack of accessibility	2 (33)	7 (47)
Lack of perceived usefulness	1 (17)	6 (40)
Lack of interoperability	0 (0)	2 (13)
Lack of attractiveness	0 (0)	1 (7)
Lack of tailored content	0 (0)	1 (7)
**Facilitators**		
Perceived usefulness	6 (100)	15 (100)
Perceived ease of use	3 (50)	2 (13)
Attractiveness	0 (0)	4 (27)
Participation of end-users during implementation	3 (50)	0 (0)
Privacy and security	1 (17)	2 (13)
Accessibility	0 (0)	2 (13)
Credibility	0 (0)	2 (13)
Content tailored to patients	0 (0)	1 (7)
Interoperability with EHR	0 (0)	1 (7)
**Individual professional**		
**Barriers**		
Lack of knowledge	1 (17)	3 (20)
Lack of motivation to change	0 (0)	1 (7)
**Facilitators**		
Positive attitude	3 (50)	10 (67)
Motivation to change	2 (33)	6 (40)
Having knowledge	2 (33)	3 (20)
**Patient**		
**Barriers**		
Lack of sufficient eHealth literacy	4 (67)	9 (60)

Negative attitude/lack of need	1 (17)	4 (27)
Negative attitude or opinion of medical professionals	1 (17)	7 (47)
**Facilitators**		
Sufficient eHealth literacy	0 (0)	4 (27)
Positive attitude/demand	1 (17)	1 (7)
**Social context**		
**Barriers**		
Negative attitude or opinion of colleagues in general	3 (50)	3 (20)
Different opinions about IT security	1 (17)	0 (0)
**Facilitators**		
Positive attitude or opinion of medical professionals	2 (33)	3 (20)
Positive attitude and opinion of colleagues in general	1 (17)	5 (33)
Different opinions about implementation	0 (0)	1 (7)
**Organizational context**		
**Barrier**		
Innovation-averse culture	3 (50)	3 (20)
Lack of resources	2 (33)	12 (80)
Lack of time and increased workload	4 (67)	4 (27)
Lack of suitable specialist staff	1 (17)	5 (33)
Adjusting organization of care processes is difficult	3 (50)	2 (13)
Structures of the organization	3 (50)	2 (13)
Change in task and new responsibilities	2 (33)	2 (13)
Organization is not ready for implementation	1 (17)	3 (20)
No strategic plan and lack of priority	0 (0)	2 (13)
**Facilitators**		
Innovation-oriented culture	1 (17)	4 (27)
Good collaboration with colleagues	2 (33)	2 (13)
Early adopters	0 (0)	3 (20)
Management support	2 (33)	6 (40)
Sufficient resources	2 (33)	4 (27)
Communication to promote the portal	2 (33)	4 (27)
Suitable specialist staff	0 (0)	2 (13)
**Economic and political context**		
**Barrier**		
Financial difficulties	4 (67)	10 (67)
Restricting laws and regulations	0 (0)	4 (27)
Third-party dependency	1 (17)	1 (7)

Lack of generic guidelines	1 (17)	0 (0)
**Facilitators**		
Supporting laws and regulations	2 (33)	3 (20)
Conducive financial arrangements	2 (33)	1 (7)
Supporting healthcare policies	0 (0)	3 (20)
Good collaboration with third parties	1 (17)	1 (7)

[a] total n=2 hospitals; total n=6 subjects

[b] total n=5 hospitals; total n=15 subjects
